# The PlexiQoL, a patient-reported outcome measure on quality of life in neurofibromatosis type 1-associated plexiform neurofibroma: translation, cultural adaptation and validation into the Dutch language for the Netherlands

**DOI:** 10.1186/s41687-024-00714-y

**Published:** 2024-03-18

**Authors:** Britt A. E. Dhaenens, Sarah A. van Dijk, Walter Taal, D. Christine Noordhoek, Anna Coffey, Stephen P. McKenna, Rianne Oostenbrink

**Affiliations:** 1 Department of General Paediatrics, Erasmus MC-Sophia Children’s Hospital, Wytemaweg 80, Rotterdam, 3015 CN the Netherlands; 2 The ENCORE Expertise Centre for Neurodevelopmental Disorders, Erasmus MC, Rotterdam, the Netherlands; 3 Department of Neurology, Erasmus MC Cancer Institute, Dr. Molewaterplein 40, Rotterdam, 3000 CA the Netherlands; 4 Galen Research Ltd, 3 Cambridge St, Manchester, UK; 5 Full Member of the European Reference Network on Genetic Tumour Risk Syndromes (ERN GENTURIS), Rotterdam, the Netherlands

**Keywords:** Neurofibromatosis type 1, Plexiform neurofibroma, Quality of life, QoL, Patient-reported outcome measure, PRO

## Abstract

**Background:**

Half of the patients with Neurofibromatosis type 1 (NF1) develop one or more tumours called plexiform neurofibromas, which can have a significant impact on Quality of Life (QoL). The PlexiQoL questionnaire is a disease-specific QoL measure for adults with NF1-associated plexiform neurofibromas. The aim of this study was to adapt and validate a Dutch version of the PlexiQoL for the Netherlands.

**Methods:**

The PlexiQoL was translated using the dual-panel methodology, followed by cognitive debriefing interviews to assess face and content validity. The psychometric properties were evaluated by administering the questionnaire on two separate occasions to a sample of adults with NF1 and plexiform neurofibromas. Feasibility was evaluated by the presence of floor/ceiling effects. Reliability was assessed by evaluating Cronbach’s alpha coefficient and test-retest reliability, using Spearman’s rank correlation coefficients. Mann-Whitney U tests were used to check for known group validity. The Nottingham Health Profile (NHP) questionnaire was used as comparator questionnaire to evaluate convergent validity.

**Results:**

The translation and cognitive debriefing interviews resulted in a Dutch version of the PlexiQoL that reflected the original concept and underlying semantic meanings of the UK English version. Forty participants completed the validation survey. The Dutch PlexiQoL demonstrated excellent internal consistency (Cronbach’s α 0.825) and test-retest reliability (Spearman correlation coefficient 0.928). The questionnaire detected differences in PlexiQoL scores between participants based on self-reported general health and disease severity. Convergent validity was confirmed for relevant NHP subsections.

**Conclusions:**

The Dutch PlexiQoL demonstrated excellent psychometric properties and can be reliably used to measure plexiform neurofibroma-related QoL in adults with NF1 in the Netherlands.

**Supplementary Information:**

The online version contains supplementary material available at 10.1186/s41687-024-00714-y.

## Background

Neurofibromatosis type 1 (NF1) is a rare hereditary disorder with a prevalence of 1 in 2.000 to 1 in 3.647 [1–4]. It is associated with a wide range of disease manifestations, such as skin lesions, cognitive impairment, and tumours of the nervous system [1, 5, 6]. Approximately 50% of the individuals with NF1 will develop one or more plexiform neurofibroma, which are tumours of the nerve sheath [6–8]. Plexiform neurofibromas can cause significant morbidity due to their size and/or location. They also are at risk of malignant transformation, which occurs in approximately 8–16% of patients with NF1 [4]. Patients with NF1-associated plexiform neurofibromas can present with a variety of symptoms such as pain, neurological deficits, disfigurement, psychological stress, and compression of vital organs [9, 10]. Therefore, plexiform neurofibromas can have a significant and lifelong impact on the patient’s Quality of Life (QoL).

The impact of a condition on QoL can be measured with Patient-Reported Outcomes (PROs). It has become increasingly important to use PROs as outcome measures in clinical practice and clinical trials for NF1-associated plexiform neurofibroma [11]. The symptoms and impact of the plexiform neurofibromas can differ significantly between patients. As such, PROs are crucial to reflect the experiences and needs of each individual [12]. They also provide meaningful insight into the effect of a treatment on the patient’s wellbeing.

PROs can be divided into generic and disease-specific measures. Generic PROs measure aspects of QoL in a general population and often do not capture disease-specific problems and symptoms [13]. There is evidence from the literature that disease-specific PROs have better psychometric properties and are more sensitive to change than generic QoL measures [14–16]. Currently, only a limited number of disease-specific PROs have been developed and validated for NF1 [17].

Heaney et al. recently developed the first QoL measure specifically for adults with NF1-associated plexiform neurofibroma: the Plexiform Neurofibromas Quality of Life (PlexiQoL) questionnaire [18]. The PlexiQoL is a needs-based QoL measure that was developed from qualitative interviews with adults with NF1 and plexiform neurofibromas. Needs-based measures are patient-centric and focus on issues that impact the ability of a patient to satisfy their human needs [19].

The aim of this study was to adapt and validate a Dutch version of the original UK English PlexiQoL for the Netherlands, to enable the use of this questionnaire in clinical practice and trials.

## Methods

### The PlexiQoL

The PlexiQoL consists of 18 items with a dichotomous response option (True / Not True). The number of times that a participant chooses ‘True’ is summed to produce a score ranging from 0 to 18, with a higher score indicating poorer QoL. The development and validation of the original PlexiQoL was conducted in the UK and US simultaneously [18]. It demonstrated excellent internal consistency (Cronbach’s α 0.90) and reproducibility (Spearman correlation coefficient 0.90), and it could distinguish between groups of patients based on general health perceptions and perceived plexiform neurofibroma severity (known groups validity) in the developmental sample.

The validation of the Dutch PlexiQoL for the Netherlands consisted of three stages: translation, assessment of face and content validity, and further psychometric evaluation (feasibility, reliability, convergent validity, and known groups validity).

### Translation

The dual panel translation methodology as recommended by Hunt et al. was employed to translate the UK English version of the PlexiQoL into Dutch [20]. This methodology emphasises the importance of achieving conceptual equivalence in the translated items to the original. This method of translation does not involve back-translation. Rather, for non-English speaking countries, two stages are conducted; a linguistic stage (to provide the initial translation into the target language) and a lay stage (where items are assessed for comprehension and ‘naturalness’ of language). This methodology has been used in all needs-based PRO adaptations [21–23]. The method was adapted slightly due to the COVID-19 pandemic: a video conference call system was used instead of performing in-person meetings. The linguistic meeting included an expert linguist who was fluent in English and Dutch, with Dutch as their native language as well as a researcher from the UK PlexiQoL development team. The role of this individual was to guide the process, and to explain the precise conceptual meaning of the items to the linguist. The purpose of this stage was to suggest translations for the instructions, items, and response categories, while ensuring that the Dutch translations captured the underlying semantic meaning behind each item. If consensus could not be reached, proposals for alternative translations of the item were sent to the lay people for consideration.

The translated version was then presented to lay people by individual interviews. The lay interviews were held with two male and three female monolingual Dutch-speaking individuals from the Netherlands, aged between 22 and 65 years, who did not have NF1, and who had low to average educational backgrounds. This criteria was to ensure that the final wording was at an appropriate level for typical patients. The lay people were recruited via a market research company in the Netherlands. During the interviews, the items of the questionnaire were assessed for comprehension and the ‘naturalness’ of the language used. In particular, participants were asked whether the phrasing of the items was acceptable or whether these should be changed to make the items more natural or simpler in Dutch, whilst maintaining the original meaning. In addition, the lay people were presented with three different translations of the response options ‘True/Not true’ to see which one was preferred, because in Dutch there are multiple ways of saying this in response to the respective question (‘does this statement apply to you’).

### Face and content validity

The purpose of the Cognitive Debriefing Interviews (CDIs) was to test the applicability, relevance and comprehensiveness of the instrument as seen by relevant patients. Ten CDIs were conducted with adults that had NF1-associated plexiform neurofibroma. It is recommended to perform five to eight interviews [24], however, in order to keep it consistent with other needs-based measures and allowing for adequate variation in age, gender and disease severity in patients, ten were conducted. The verbal probing technique was utilised [25]. Patients were asked to participate in the CDIs when they visited the Neurology outpatient clinic of the Erasmus Medical Centre in Rotterdam, the Netherlands. Participants had to meet the revised diagnostic criteria for NF1 [26] and have at least one plexiform neurofibroma. All invited participants agreed to participate (response rate 100%). In the one-to-one, semi-structured interviews, participants were asked to complete the questionnaire in the presence of a trained interviewer using a video call system. As such, the interviewer could observe difficulties or hesitation when completing specific items. After completing the questionnaire, scripted verbal probes were used to ask the participants whether they considered the items relevant, applicable, and comprehensible and if they believed that any important aspects of their experience with NF1-associated plexiform neurofibroma had been omitted. This was combined with additional unscripted probes for items where the interviewees showed difficulties or hesitation when completing the questionnaire.

### Further psychometric evaluation

The psychometric properties of the Dutch PlexiQoL were evaluated through a postal survey. Adults who met the revised diagnostic criteria for NF1 [26] and who had one or more plexiform neurofibroma were recruited when they visited the Neurology outpatient clinic of the Erasmus Medical Centre from July 2022 through April 2023. This was a new sample of participants who had not taken part in the previous CDIs. The PlexiQoL was administered to the participants on two occasions, with 14 days between the two administrations.

At the first administration, the Nottingham Health Profile (NHP) was included as a comparator questionnaire [27]. The NHP is a generic health profile questionnaire that provides an indication of a patient’s perceived emotional, social and physical health problems. It consists of 38 items that cover six subsections: physical mobility, pain, energy level, emotional reactions, sleep and social isolation. For each section, scores range from 0 to 100, with higher scores indicating greater perceived distress. In addition, a form was included to collect basic demographic information, including sex, age, the self-perceived general health and severity of the plexiform neurofibroma (both rated on a 4-point Likert scale), current treatment for the plexiform neurofibroma, and other health problems. The second administration consisted of the PlexiQoL and a shortened version of the demographic form, inquiring about the perceived general health, plexiform neurofibroma severity, and other health problems.

Additional clinical information was extracted from the electronic health records of the participants. Extracted items consisted of the location of the plexiform neurofibroma, the presence of certain plexiform neurofibroma-related complications (pain which required drug intervention, neurological deficits, and disfigurement), other NF1-related disease manifestations (osseous lesions, optic pathway glioma (OPG), other brain glioma, malignant peripheral nerve sheath tumour), mutation type (familial/de novo), and the presentation of NF1 (generalised/segmental).

### Statistical analysis

The statistical analyses were conducted using SPSS version 28.0. Non-parametric tests were used given the ordinal nature and non-normal distribution of the data. PlexiQoL and NHP scores were computed according to their respective scoring instructions. For the descriptive analyses the mean, standard deviation (SD) and range were calculated for continuous variables, and count and frequency for categorical variables. Mann-Whitney U or Kruskal-Wallis tests were used to see if there were differences in PlexiQoL scores between participants grouped by demographic factors. The feasibility of the PlexiQoL was assessed by determining the response rate, percentage of missing answers, and the presence of floor and/or ceiling effects. Floor and ceiling effects were considered significant if ≥ 15% of the participants scored the lowest or highest absolute value on the questionnaire [28]. For all analyses, significance was based on a two-sided p-value of < 0.05.

Cronbach’s α was calculated to evaluate the internal consistency, which measures the extent to which the items of the questionnaire are interrelated. A Cronbach’s α of ≥ 0.70 is considered adequate [29]. The test-retest reliability of the Dutch PlexiQoL was assessed by calculating the Spearman’s rank correlation coefficients between the PlexiQoL scores of the first and second administration for each participant. A value of ≥ 0.75 indicates that the questionnaire has adequate test-retest reliability, showing low levels of random measurement error [30].

Convergent validity was assessed by the Spearman rank correlation coefficients between the scores of the PlexiQoL and the scales of the comparator measure (NHP) that measure the same or related constructs in QoL. It was hypothesized that the PlexiQoL score would correlate significantly with all sections of the NHP, with exception of the ‘Pain’ and ‘Sleep’ section, based on the items contained in the PlexiQoL. Mann-Whitney U tests were used to investigate whether the PlexiQoL would be able to discriminate between groups of participants based on self-reported health perceptions, plexiform neurofibroma severity, treatment for plexiform neurofibroma yes/no, the presence of other health problems, and the presentation of NF1 (general vs. segmental) (known groups validity). Effect sizes were assessed using Cohen’s *d*; 0.20 ≤ *d* < 0.50 were considered small, 0.50 ≤ *d* < 0.80 as medium, and *d* ≥ 0.80 were considered large [31].

## Results

### Translation

Overall, the linguist found the instructions and most of the items straightforward and easy to translate. Some items were more difficult to translate and were passed on to the lay people and CDIs for further discussion. For example, ‘I am very self-conscious about the way I look’ proved difficult to translate into Dutch due to the direct translation being too harsh compared to the original English.

Following the interviews with the lay people minor changes were made to five out of eighteen items to make them clearer and more natural in Dutch, with the participants suggesting some variations. Overall, the participants found the questionnaire well-written and easy to understand. Some items were passed to the CDIs, to check the appropriateness of the translation, and to check the participants’ understanding of these items.

### Face and content validity

Seven of the ten CDI participants were female, and the participants’ ages ranged from 20 to 59 years old. The mean time to complete the PlexiQoL questionnaire was 4 min (range 2–8 min). Overall, the participants found the questionnaire clear and easy to complete, with all the items being relevant to their disease. The participants confirmed their understanding of the items that were passed to them from the lay stage. Minor changes were made to some items following suggestions made by the participants of the CDIs. For example, the item ‘The quality of my relationships is affected’ was amended in Dutch to better transmit the reference to family and friends. Likewise, the item ‘I avoid intimate situations’ was modified to better convey that this includes situations with family and friends. Some participants preferred to have the phrase ‘as you are aware’ removed from the instructions, as not all patients will be familiar with the term plexiform neurofibroma. The instructions were changed accordingly (original text: ‘As you are aware, plexiforms are tumours that grow on nerves underneath the skin’).

### Psychometric evaluation

The response rate for the first administration was 73% (40/55 surveys completed). Forty adults with NF1 and plexiform neurofibromas participated in the validation of the PlexiQoL, of whom 39 also completed the second administration. The demographic and disease information of the participants can be seen in Table 1. Most participants self-perceived their general health as ‘good’ (63%), and the severity of their plexiform neurofibroma as ‘moderate’ (65%). The plexiform neurofibroma were seen in a variety of locations, but plexiform neurofibroma in the head/neck and limbs were the most common. More than half (60%) of the participants experienced pain, neurological deficits or disfigurement due to their plexiform neurofibroma. 33% of the participants received treatment for their plexiform neurofibroma (pain medication or MEK-inhibitors).

**Table 1:** *Demographic and disease information of postal survey sample (n* = 40)

	Mean (SD) or range	n	%
Participant characteristics			
Sex (male)		15	38
Age (years)			
Mean (SD)	39.7 (14.8)		
Range	19–67		
Marital status			
Married / Living as married		21	53
Single		19	48
Employment status (*n* = 39)			
Employed (full-time and part-time)		27	69
Retired		2	5
Long term sick leave		4	10
Student		3	8
Unemployed		3	8
**Disease information**			
Self-reported general health			
Poor		1	3
Fair		12	30
Good		25	63
Very good		2	5
Self-reported severity of PN			
Mild		6	15
Moderate		26	65
Severe		7	18
Very severe		1	3
Treatment for PN		13	33
Other health problems present (self-reported)		15	38
**Other clinical characteristics**			
De novo NF1 mutation		30	77
Generalised NF1 presentation		35	88
PN location			
Spinal		7	18
Head/neck		10	25
Thorax		1	3
Abdomen/pelvis		6	15
Limb		16	40
PN-related symptoms			
Pain		13	33
Neurological deficits		6	15
Disfigurement		5	13
No symptoms		16	40

Other NF1-related manifestations were present only in a small number of participants: one participant had a history of OPG, one had a low grade non-OPG brain glioma and four had an osseous lesion. Malignant peripheral nerve sheath tumours were not observed in this participant sample.

We found no significant differences in PlexiQoL scores between participants grouped by age (above and below median age (38 years)), sex, marital status or employment status (Additional File 1).

### Feasibility and reliability

The scores of the PlexiQoL (both at first and second administration) and the NHP questionnaire can be seen in Table 2. There were no instances of missing values. For the PlexiQoL there were no floor effects, and the observed ceiling effects were not significant. Significant floor effects were observed on all six NHP subsections, as well as one ceiling effect on the ‘Energy Scale’ subsection. Participants reported the greatest perceived distress on the ‘Sleep Scale’ compared to the other NHP subsections.

The PlexiQoL showed high internal consistency at both administrations (Table 2). The item-total correlations for each item are presented in Additional File 2. We found a correlation coefficient of 0.928 between the first and second administration, indicating excellent test-retest reliability (p-value < 0.001).

### Convergent validity

The PlexiQoL score showed strong correlation with the NHP ‘Emotional Reactions’ and ‘Social Isolation’ subsections (Table 3). There was a moderate correlation between the ‘Energy Scale’ and the ‘Physical Mobility’ subsections. The ‘Pain Scale’ and the ‘Sleep Scale’ did not significantly correlate with the PlexiQoL score.

**Table 2:** Descriptive statistics, feasibility and reliability for the PlexiQoL and NHP. IQR = Inter-quartile range

	n	Median (IQR)	Min - Max	Scoring minimum (%)	Scoring maximum (%)	Cronbach’s α
**PlexiQoL (1st** **administration)**	40	5.0 (2.0–8.0)	0–15.0	2 (5.0)	0	0.825
**PlexiQoL (2nd administration)**	39	4.0 (2.0–7.0)	0–15.0	3 (7.7)	0	0.844
**NHP**						
***Energy Scale***	40	0.0 (0.0–66.7)	0–100	21 (52.5)	9 (22.5)	
***Pain Scale***	40	12.5 (0.0–62.5)	0–100	16 (40.0)	3 (7.5)	
***Emotional Reactions***	39	11.1 (0.0–33.3)	0–100	16 (41.0)	1 (2.6)	
***Sleep Scale***	39	20.0 (0.0–40.0)	0–100	13 (33.3)	1 (2.6)	
***Social Isolation***	40	0.0 (0.0–20.0)	0–100	24 (60.0)	1 (2.5)	
***Physical Mobility***	40	0.0 (0.0–21.9)	0–87.5	25 (62.5)	1 (2.5)	

**Table 3:** Correlation coefficients between PlexiQoL scores and NHP scales

	PlexiQoL
NHP	
***Energy Scale***	0.584*
***Pain Scale***	0.378
***Emotional Reactions***	0.793*
***Sleep Scale***	0.391
***Social Isolation***	0.831*
***Physical Mobility***	0.567*

*p < 0.001

### Known group validity

For the known group validity analysis, the categories for general health perceptions and plexiform neurofibroma severity were grouped into two categories due to the small number of participants in the individual groups. Significant differences in PlexiQoL scores were observed between participants regarding their self-reported general health and plexiform neurofibroma severity (with effect sizes of 0.48 and 0.42, respectively) (Table 4). We found no significant differences between participants with and without treatment, participants with and without other health problems, and participants with generalised versus segmental NF1.

**Table 4:** Median PlexiQoL scores by known group factors

	n (40 total)	Median PlexiQoL score (IQR)
**Self-reported general health**		
Poor & Fair	13	8.0 (4.0)
Good & Very Good	27	3.0 (4.0)
***p-value (effect size)***		**0.002 (0.48)**
**Self-reported severity of PN**		
Mild & Moderate	32	4.0 (5.0)
Severe & Very Severe	8	9.5 (4.5)
***p-value (effect size)***		**0.009 (0.42)**
**Treatment for PN**		
Yes	13	8.0 (8.5)
No	27	4.0 (5.0)
***p-value (effect size)***		0.073 (0.29)
**Presence of other health problems**		
Yes	15	7.0 (8.0)
No	25	4.0 (5.5)
***p-value (effect size)***		0.099 (0.27)
**Presentation of NF1**		
Generalised	35	5.0 (6.0)
Segmental	5	2.0 (3.5)
***p-value (effect size)***		0.083 (0.27)

## Discussion

By utilising the dual-panel methodology, the translation of the Dutch PlexiQoL for the Netherlands resulted in an equivalent version which reflects the same concept and semantic meanings as the original UK English. The cognitive debriefing interviews confirmed that all items of the PlexiQoL were relevant to adults with NF1-associated plexiform neurofibroma. After some minor changes, all items were considered clear and easy to understand. The Dutch version showed excellent psychometric properties, which are comparable to those of the original English version [18].

The high internal consistency of the Dutch PlexiQoL at both administrations (Cronbach’s α of 0.825 and 0.844 in the present study, 0.90 in the original PlexiQoL) indicates that the items are consistent and measure the same construct (QoL). The excellent test-retest reliability (correlation coefficient of 0.93, compared with 0.90 of the English PlexiQoL) demonstrates that the Dutch version of the PlexiQoL will produce consistent results with a low chance of random measurement errors. A two-week interval was used, which is considered an adequate margin to avoid both recall bias and clinical improvement or deterioration [28]. Regarding convergent validity, the Dutch PlexiQoL significantly correlated as expected with four subsections of the NHP, indicating that plexiform neurofibroma-related QoL is closely entwined with physical, emotional, and social wellbeing.

Similar to the English PlexiQoL, there was also evidence of known group validity, as the questionnaire was able to detect meaningful differences in QoL between participants with distinct self-perceptions of plexiform neurofibroma severity and general health. No significant differences in PlexiQoL scores were found between participants based on demographic factors, suggesting that these factors do not substantially influence plexiform neurofibroma-related QoL.

No floor and ceiling effects of significance were observed for the PlexiQoL, in contrast to the NHP questionnaire, which displayed significant floor effects on all subsections. This finding suggests that the NHP questionnaire is less suited to measure aspects of QoL in adults with NF1, because it cannot adequately detect (small) differences in QoL between individuals who have a higher QoL. This finding underlines the importance of using disease-specific rather than generic QoL measures, as disease-specific measures may be more suited to detect differences in QoL between individuals with NF1.

Surprisingly, no differences were observed in PlexiQoL scores between participants with and without treatment, despite that more severe cases of NF1-associated plexiform neurofibroma tend to be treated with pain medication or MEK-inhibitors. In this study, participants who received treatment perceived their disease as ‘severe/very severe’ significantly more often than the non-treated participants (results not shown). The absence of a significant difference in PlexiQoL scores could be explained by the small number of treated patients: only thirteen participants received treatment, of whom five were treated with a MEK-inhibitor. Treatment with MEK-inhibitors in adults with NF1-associated plexiform neurofibroma is uncommon, as they have not been approved by the European Medicines Agency (EMA) in this specific patient population. It would be interesting to see if differences in PlexiQoL scores could be detected between (MEK-inhibitor) treated patients and non-treated patients in a larger study sample.

There was no significant correlation observed with the ‘Sleep Scale’ and ‘Pain Scale’ of the NHP, as predicted, since the PlexiQoL questionnaire does not contain any items on sleep and pain. However, pain is a common morbidity of NF1-associated plexiform neurofibroma and it often has a considerable impact on the daily functioning of adults with NF1-associated plexiform neurofibroma [9, 10, 32]. By omitting questions on the influence of pain, the PlexiQoL could miss an important aspect of plexiform neurofibroma-related QoL. Further administration of the questionnaire in participants with plexiform neurofibroma-related pain would be required to study this further. It should be noted that the English PlexiQoL did correlate significantly with both the ‘Sleep’ and ‘Pain’ section of the NHP. While the Dutch PlexiQoL did not show significant correlations with these sections, there was a positive trend. The lack of significance could be explained by the small sample size which may be insufficient to detect a meaningful difference.

There is a distinction between needs-based QoL and Health-related Quality of Life (HRQoL). The PlexiQoL is a needs-based QoL measure, which focusses on the impact of a condition on the ability of a patient to satisfy their needs. HRQoL questionnaires collect information on factors that are directly influenced by the presence of symptoms and treatment. HRQoL measures tend to be especially relevant to clinicians and researchers, while needs-based QoL measures are considered more patient-centric. To adequately measure multiple types of outcomes, the use of a combination of needs-based and HRQoL measures should be considered. The PlexiQoL is the first patient-derived and needs-based QoL measure designed specifically for adults with NF1-associated plexiform neurofibroma and could be a valuable tool for use in clinical trials and clinical practice, especially when combined with a HRQoL measure.

The main limitation of this study is the small sample size. Although the Erasmus Medical Center is one of the main expertise centres in the Netherlands for NF1, providing care for more than 1000 adult NF1 patients, it was difficult to enrol a larger number of participants with this rare disease. This has an impact on the power of the study and may influence the significance of observed differences in PlexiQoL scores between groups (e.g., between patients with and without treatment). The original development and validation study of the PlexiQoL did include a large sample size of 273 participants [18]. Given that the psychometric properties of the Dutch and original English version are comparable, we can conclude that the Dutch version of the PlexiQoL is reliable for future use. In addition, this study was not designed to assess the PlexiQoL’s responsiveness to change. Outcome measures in clinical trials must be sensitive to change over time in order to adequately measure a treatment effect. The excellent test-retest reliability indicates that the PlexiQoL is not prone to random measurement errors. It now should be studied how well the PlexiQoL can detect (small) differences in QoL over time, in this chronic condition that tends to progress slowly with variable associated morbidity. Lastly, during the translation process, no backward translation was performed. Generally, a backward translation is recommended in the adaptation process for PROs [24]. However, as the PlexiQoL is a needs-based PRO, the items of the questionnaire are based directly on the interview transcripts of the patient interviews. As such, they are kept as close to the patients original wording as possible to maintain content validity, and therefore they are largely colloquial. As a back translation would miss these nuances, it was decided to not perform one, in accordance with the adaptation process of other needs-based PROs [21–23].

## Conclusions

The Dutch version of the PlexiQoL for the Netherlands demonstrated excellent psychometric properties. It can be reliably used to measure aspects of plexiform neurofibroma-related QoL in adults with NF1 and is able to distinguish between adults based on self-reports of general health and plexiform neurofibroma severity. This new Dutch version can now be used in the Netherlands in clinical practice and trials to assess the QoL of adults who have NF1-related plexiform neurofibromas.

### Electronic supplementary material

Below is the link to the electronic supplementary material.

Supplementary Material 1

Supplementary Material 2

## Data Availability

The datasets generated and/or analysed during the current study are not publicly available due to sensitivity of the data and the risk of compromising the individual privacy of the participants. The analysed data is only available from the corresponding author on reasonable request. The Dutch PlexiQoL is a copyrighted instrument and is available to license on request from Galen Research.

## Abbreviations

CDI: Cognitive Debriefing Interviews
HRQoL: Health Related Quality of Life
IRB: Institutional Review Board
NHP: Nottingham Health Profile
NF1: Neurofibromatosis Type 1
PlexiQoL: Plexiform Neurofibromas Quality of Life questionnaire
PRO: Patient-Reported Outcome
QoL: Quality of Life
UK: United Kingdom
USA: United States of America
