# Supplementary material for: The PlexiQoL, a patient-reported outcome measure on quality of life in neurofibromatosis type 1-associated plexiform neurofibroma: translation, cultural adaptation and validation into the Dutch language for the Netherlands
Source: J Patient Rep Outcomes. 2024 Mar 18;8:33. doi: 10.1186/s41687-024-00714-y (PMC10948685; doi:10.1186/s41687-024-00714-y)
Supplement: Supplementary file 1 — Supplementary Material 1 [file 41687_2024_714_MOESM1_ESM.pdf]

Supplementary file 1 - Differences in PlexiQoL scores based on demographic factors

	n	Median PlexiQoL score (IQR)
**Sex**	40	
Male	15	4.0 (6.0)
Female	25	5.0 (6.0)
p-value		0.767
**Age (median = 38)**	40	
Below median	20	5.0 (5.8)
Above median	20	5.0 (6.0)
p-value		0.495
**Marital status**	40	
Married/Living as married	21	4.0 (5.5)
Divorced/Separated	1	8.0 (0.0)
Single	18	6.0 (9.0)
p-value		0.305
**Employment status**	40	
Full-time	19	5.0 (5.0)
Part-time	8	5.0 (6.0)
Retired	1	1.0 (0.0)
Long term sick leave	4	8.0 (10.8)
Student	3	3.0 (-)
Homemaker	1	5.0 (0.0)
Retired due to sickness	1	8.0 (0.0)
Unemployed	2	12.0 (-)
p-value		0.375
