# Supplementary material for: The PlexiQoL, a patient-reported outcome measure on quality of life in neurofibromatosis type 1-associated plexiform neurofibroma: translation, cultural adaptation and validation into the Dutch language for the Netherlands
Source: J Patient Rep Outcomes. 2024 Mar 18;8:33. doi: 10.1186/s41687-024-00714-y (PMC10948685; doi:10.1186/s41687-024-00714-y)
Supplement: Supplementary file 2 — Supplementary Material 2 [file 41687_2024_714_MOESM2_ESM.pdf]

Supplementary file 2 - Item-total correlations for the PlexiQoL

Item-total correlations and the Cronbach's alpha if the item were to be deleted for each item of the PlexiQoL at the first administration.

Item	Scale Mean if Item Deleted	Scale Variance if Item Deleted	Corrected Item-Total Correlation	Cronbach's Alpha if Item Deleted
PlexiQoL Q1	5.21	13.738	0.476	0.813
PlexiQoL Q2	5.24	13.861	0.459	0.814
PlexiQoL Q3	5.32	13.898	0.559	0.811
PlexiQoL Q4	5.39	14.840	0.317	0.822
PlexiQoL Q5	5.16	13.055	0.655	0.802
PlexiQoL Q6	4.82	14.317	0.240	0.827
PlexiQoL Q7	4.79	14.819	0.106	0.835
PlexiQoL Q8	5.18	14.803	0.128	0.832
PlexiQoL Q9	5.37	14.780	0.280	0.823
PlexiQoL Q10	5.08	13.048	0.611	0.804
PlexiQoL Q11	5.21	13.522	0.548	0.809
PlexiQoL Q12	5.24	13.645	0.533	0.810
PlexiQoL Q13	5.18	13.776	0.443	0.815
PlexiQoL Q14	5.03	14.080	0.296	0.824
PlexiQoL Q15	5.29	14.049	0.454	0.815
PlexiQoL Q16	5.03	13.594	0.433	0.816
PlexiQoL Q17	5.03	12.999	0.608	0.804
PlexiQoL Q18	5.05	13.673	0.416	0.817
